# Treatment Limitation Decisions in Critically Ill Patients With a Malignancy on the Intensive Care Unit

**DOI:** 10.1177/0885066620948453

**Published:** 2020-08-13

**Authors:** Esther N. van der Zee, Jelle L. Epker, Jan Bakker, Dominique D. Benoit, Erwin J. O. Kompanje

**Affiliations:** 1 Department of Intensive Care, 6993 Erasmus MC—University Medical Center Rotterdam, the Netherlands; 2 Department of Pulmonology and Critical Care, New York University NYU Langone Medical Center, New York, NY, USA; 3 Department of Pulmonology and Critical Care, Columbia University Medical Center, New York, NY, USA; 4 Department of Intensive Care, Pontificia Universidad Católica de Chile, Santiago, Chile; 5 Department of Intensive Care, 60200 Ghent University Hospital, Ghent, Belgium

**Keywords:** malignancy, neoplasm, do-not-resuscitate, treatment limitation, critical care, ICU

## Abstract

**Background:**

Treatment limitation decisions (TLDs) on the ICU can be challenging, especially in patients with a malignancy. Up-to-date literature regarding TLDs in critically ill patients with a malignancy admitted to the ICU is scarce. The aim was to compare the incidence of written TLDs between patients with an active malignancy, patients with a malignancy in their medical history (complete remission, CR) and patients without a malignancy admitted unplanned to the ICU.

**Methods:**

We conducted a retrospective cohort study in a large university hospital in the Netherlands. We identified all unplanned admissions to the ICU in 2017 and categorized the patients in 3 groups: patients with an active malignancy (study population), with CR and without a malignancy. A TLD was defined as a written instruction not to perform life-saving treatments, such as CPR in case of cardiac arrest. A multivariate binary logistic regression analysis was used to identify whether having a malignancy was associated with TLDs.

**Results:**

Of the 1046 unplanned admissions, 125 patients (12%) had an active malignancy and 76 (7.3%) patients had CR. The incidence of written TLDs in these subgroups were 37 (29.6%) and 20 (26.3%). Age (OR 1.03; 95% CI 1.01 -1.04), SOFA score at ICU admission (OR 1.11; 95% CI 1.05 -1.18) and having an active malignancy (OR 1.75; 95% CI 1.04-2.96) compared to no malignancy were independently associated with written TLDs. SOFA scores on the day of the TLD were not significantly different in patients with and without a malignancy.

**Conclusions:**

This study shows that the presence of an underlying malignancy is independently associated with written TLDs during ICU stay. Patients with CR were not at risk of more written TLDs. Whether this higher incidence of TLDs in patients with a malignancy is justified, is at least questionable and should be evaluated in future research.

## Introduction

Invasive life-saving treatments, provided at an intensive care unit (ICU), have been increasingly offered to patients with potentially lifespan limiting conditions, such as a malignancy.^1–4^ Even an increase in such treatments in the final stages of life of these patients has been reported.^3^ These therapies should not automatically be considered disproportional; however, the treatment intensity should remain proportional to the expected outcome.^1^ In 2 large multicenter studies, perceptions of inappropriate ICU care were frequently reported by clinicians.^1,5^

Approximately 30% of patients requiring cardiopulmonary resuscitation (CPR) after an in-hospital cardiac arrest (IHCA) survived the hospital admission,^6^ the survival of patients with return of spontaneous circulation admitted to the ICU is up to 50%.^7^ In contrast, hospital survival of patients with a malignancy requiring CPR after IHCA was significantly lower (5-10%).^8,9^ Therefore, CPR can in certain circumstances be seen as a traumatic, undignified and inappropriate medical intervention,^10,11^ often with prolonged hospitalization and invasive treatment as result.^12,13^ A decision to withhold invasive life-saving treatments in case of a medical emergency, such as CPR during a circulatory arrest, is called a treatment limitation decision (TLD) and is part of routine hospital practice.^9^ When properly executed, a written TLD is a useful method to ensure that patient’s preferences regarding CPR or other invasive life-saving treatments are honored.^14–16^

However, ICU patients often lack decision-making capacity^17,18^ and advanced directives are often lacking or unclear.^19-21^ Cheveaux et al. showed that “having a malignancy” is associated with more do-not-resuscitate decisions at the ward, of which more than half were made by the medical team.^22^ TLDs on the ICU can be challenging, especially in patients with a malignancy. Up-to-date literature regarding treatment limitation decisions in critically ill patients with a malignancy admitted unplanned to the ICU is scarce, despite rapid evolution of diagnostics and cancer treatments. The number of patients with a malignancy admitted unplanned to the ICU has been increasing, and mortality rates in these patients are higher than in unplanned ICU patients without a malignancy.^4,23^ A higher incidence of written TLDs in critically ill patients with a malignancy than in patients without a malignancy may be justified, especially in patients with a poor short-term prognosis. In addition, TLDs may be justified when used for minimizing inappropriate invasive life-saving treatments in case of deterioration in this vulnerable patient population. On the other hand, in case of similar severity of illness between patients with and without a malignancy, the presence of an underlying malignancy should not automatically result in TLDs.

The primary objective of this study was to compare the incidence of written TLDs between patients with an active malignancy, patients with a malignancy in their medical history and patients without a malignancy admitted unplanned to the ICU. The secondary objective was to identify factors independently associated with written TLDs in patients with a malignancy.

## Methods

We conducted a retrospective cohort study in a large university hospital ICU in the Netherlands (Rotterdam). By using our hospital electronic database, we identified all unplanned admissions to the ICU in the year 2017. Our ICU is a mixed ICU in a tertiary referral university hospital. We categorized the patients in 3 groups: patients with an active malignancy (study population), patients with a malignancy in their medical history (complete remission, CR) and patients without a malignancy. The study was approved by the ethical committee of our institute (MEC-2018-1172).

A metastatic solid tumor was defined as the presence of cancer cells present in distant organs or distant lymph nodes, determined by using the medical reports of hematologists and oncologists. Patients with treatment for a malignancy in their medical history and no signs of recurrence were defined as complete remission (CR). We defined CR as no detectable malignancy based on the information available in the electronic database, medical reports and letters. We divided complete remission in CR < 1 year and CR > 1 year, in order to examine the influence of very recent CR compared to longer existent CR in written TLDs. Late deleterious effects of cancer therapies can occur even decades after completion of the cancer treatment,^24–28^ we therefore did not exclude patients with a malignancy, even patients with a malignancy in their distant past.

Patients with a diagnosis of a non-melanoma skin malignancy (squamous-cell skin cancer or basal-cell carcinoma) were excluded because of the relatively favorable prognosis of these tumors, usually without life threatening complications. Similarly, we also excluded patients with a premalignant condition, such as colon polyps.

A treatment limitation decision was defined as a written instruction not to perform cardiopulmonary resuscitation in case of a cardiac arrest, or a written instruction not to perform other life-saving treatments, such as tracheal intubation and mechanical ventilation in case of respiratory insufficiency or renal replacement therapy in case of kidney insufficiency. An actual withholding or withdrawal of ICU treatment during the ICU admission was not mandatory to meet the definition, only the written instruction. New written TLDs or an extension of an existent of a written TLD during ICU admission were collected. Furthermore, the reasons for the TLDs were collected.

ICU and patient characteristics were collected. The comorbidity of the patients was measured by using the Charlson Comorbidity Index (CCI).^29^ The Eastern Cooperative Oncology Group (ECOG) Performance Status was used to assess performance status in the month to 14 days prior to the ICU admission.^30^ To evaluate the extent of the acute critical illness of the patients at ICU admission, the Sequential Organ Failure Assessment (SOFA) score was used.^31^ Unplanned ICU admissions were defined as medical admissions and postoperative admission after emergency surgery. Readmissions were defined as a new ICU admission within 30 day after discharge from the ICU.

The primary objective of this study was to compare the incidence of written TLDs between patients with an active malignancy, patients with CR and patients without a malignancy admitted unplanned to the ICU. The secondary objective was to identify factors independently associated with written TLDs in patients with an active malignancy.

### Statistical Analysis

Descriptive statistics were used to describe baseline and ICU characteristics. Categorical variables are reported as numbers with percentage. Continuous variables are reported as mean and standard deviation for normally distributed data, or, in case of a skewed distribution, median with 25th–75th interquartile range (IQR).

#### Primary outcome

In order to analyze differences in baseline characteristics between patients with and without a malignancy with a new written TLD, we used Pearson’s Chi-square tests or the Fisher’s exact tests for categorical variables and Independent Samples T-Tests (normal distribution) or the Mann-Whitney U tests (skewed distributions) for continuous variables. A statistical test with a 2 tailed p value ≤ 0.05 was considered as significant.

To explore whether having a malignancy was independently associated with a written TLD, a univariate logistic regression analysis was used including the following variables: malignancy status (i.e. no malignancy, active malignancy, CR and both active and CR), age, gender and SOFA score at admission. Subsequently, variables with a p-value < 0.2 in the univariate analysis were analyzed in a multivariate binary logistic regression analysis. Odds ratios (OR) and p-values of these variables are presented.

#### Secondary outcome

To identify factors associated with written TLDs in the study population, a univariate binary logistic regression analysis was performed including the following variables: age, gender, marital status, body mass index, CCI, ECOG Performance Status, malignancy type, metastatic disease, admission reason, readmission, SOFA score and sepsis. Subsequently, variables with a p-value <0.2 in the univariate analysis were evaluated in a multivariate binary logistic regression analysis. Given the clinical relevance of CCI, type of malignancy and admission reason, these factors were forced into the multivariate model. Odds ratios (OR) and p-values of these variables are presented.

## Results

During the study period, 2486 patients were admitted to our ICU, of which 1046 (42%) unplanned admissions. Of these unplanned admissions, 125 (12%) patients were diagnosed with an active malignancy (study population) and 76 (7.3%) patients with CR. The baseline characteristics of the study population are shown in Table 1. The majority of the study population was diagnosed with a solid tumor (80.8%). The different types of the malignancies are shown in Appendix A. Written TLDs were made in 30 (24%) of the patients before ICU admission, the majority were made by the medical team for medical reasons (83.3%, Table 1). ICU admission characteristics are shown in Table 1 as well. During ICU stay, a written TLD was made in another 37 patients (29.6%). All of the written TLDs were made by the medical team, 2 decisions included explicit patient’s wishes (5.4%).

**Table 1.** Characteristics and Outcome Study Population.

Baseline characteristics	Study population *(n = 125)*
Age^a^	66 [59-73]
Male	84 (67.2%)
BMI in kg/m^2 b, c^	24.0 {21.8-27.1}
Marital status	
Married	87 (69.6%)
Without partner	30 (24.0%)
Other	7 (5.6%)
Unknown	1 (0.8%)
Comorbidity	
CCI ^a, d^	4 [3-6]
Age-adjusted CCI ^a, d^	6 [4-8]
Patients with a solid malignancy	101 (80.8%)
Patients with a hematological malignancy	21 (16.8%)
Patients with solid and hematological malignancy	1 (0.8%)
Patients with unknown type	2 (1.6%)
Patients with metastatic solid malignancy	33 (26.4%)
TLD^f^ before ICU^e^ admission	30 (24.0%)
Reasons TLD^f^	
Medical reasons	25 (83.3%)
Wish of patient	3 (10%)
Combination 1 and 2	2 (6.7%)
**Characteristics ICU^e^ admission**	
Admission reason	
Respiratory failure	29 (23.2%)
Post-operative: emergency operation	20 (16%)
Sepsis	15 (12%)
Post cardiopulmonary resuscitation	5 (4%)
Neurological	10 (8%)
Other	25 (20%)
Combination of reasons	21 (16.8%)
Readmissions	32 (25.6%)
Readmission < 2 days	6 (4.8%)
SOFA value ^a, g^	7 [5 -10]
Mechanically ventilated	83 (66.4%)
Vasopressors	90 (72%)
Renal Replacement Therapy	27 (6.8%)
Sepsis	54 (43.2%)
**Outcome**	
TLD^f^	37 (29.6%)
DNR^f^	23 (18.4%)
DNR and other limitations	10 (8.0%)
Only other limitations	4 (3.2%)
Reasons TLD^f^	
Medical reasons	35 (94.6%)
Wish of patient	0 (0%)
Combination of 1 and 2	2 (5.4%)
Medical reasons	
Multiple organ failure	14 (37,8%)
Poor neurological prognosis	3 (8.1%)
(Incurable) Malignancy	16 (43.2%)
Progression critical illness despite maximal medical support	17 (45.9%)
Other	7 (18.9%)
Withdrawal / withholding treatment ICU^e^	41 (32.8%)
ICU^e^ survival	85 (68%)
Hospital survival	70 (56%)
Length of stay^a, h^	3 [1-10]

^a^ Data are displayed as median with 25th and 75th percentile

^b^ BMI; Body Mass Index

^c^ A logarithmic transformation was performed, original data was not normally distributed

^d^ CCI; Carlson Comorbidity Index

^e^ ICU; Intensive Care Unit

^f^ DNR; Do-Not-Resuscitate

^g^ SOFA; Sequential Organ Failure Assessment score (SOFA score)

^h^ In days

Patients with a malignancy who received a written TLD during ICU stay were significantly older than patients without a malignancy who received a written TLD, but SOFA scores on the day of the TLD were not significantly different in patients with and without a malignancy (Table 2).

**Table 2.** Characteristics Patients With Treatment Limitations Received During ICU Stay: ICU Population Without Malignancy vs. Study Population.

	General ICU^a^ population (n = 146)	Study population (n = 37)	P-value^b^
Age^c^	64 [54-72]	68 [61-77]	0.02*
Male	103 (62%)	24 (64.9%)	0.75
SOFA^d^ at ICU admission	9 [7-11]	8 [6-11]	0.34
SOFA^d^ at TLD	9 [8-14]	9 [7-12]	0.33
Day TLD^e^	2 [1-6]	4 [1-8]	0.27

^a^ ICU; Intensive Care Unit

^b^ P- value; probability value, a p-value of < 0.05 was considered statistically significant, marked by an Asterisk *

^c^ Data are displayed as median with 25th and 75th percentile

^d^ SOFA; Sequential Organ Failure Assessment score (SOFA score)

The univariate binary logistic regression analysis showed a higher incidence of written TLDs during ICU stay in patients with an active malignancy than in patients without a malignancy (OR 1.97; 95% CI 1.29-3.01; Table 3). Having CR was not associated with a written TLD. Age and severity of illness at ICU admission (SOFA score) were associated with written TLDs as well.

**Table 3.** Univariate Analysis for Factors Associated With TLD During ICU Stay in Patients With and Without a Malignancy.

Variable	Patient cases	Treatment limitation	OR^a^	95% CI^b^	P-value^c^
No malignancy (ref)	825 (78.9%)	145 (17.6%)	–	–	–
Malignancy (active or CR^d^)	221 (21.1%)	63 (28.5%)	1.87	1.33-2.63	<0.001*
Active malignancy	125 (12.0%)	37 (29.6%)	1.97	1.29-3.01	0.002*
CR^d^ <1 yr	18 (1.7%)	4 (22.2%)	1.34	0.44-4.13	0.61
CR^d^ >1 yr	58 (5.5%)	16 (27.6%)	1.79	0.98-3.27	0.06
Active malignancy + CR^d^	16 (1.5%)	5 (31.3%)	2.13	0.73-6.23	0.17
Age	–	–	1.02	1.01 -1.03	<0.001*
Gender	661 (63.2%)	131 (19.8%)	1.01	0.74 -1.39	0.94
SOFA at ICU admission	–	–	1.10	1.04 -1.16	0.001*

^a^ OR; Odds ratio

^b^ CI; confidence interval

^c^ P- value; probability value, a p-value of < 0.05 was considered statistically significant, marked by an Asterisk *

^d^ CR; Complete remission

Age (OR 1.03; 95% CI 1.01 -1.04), SOFA score at ICU admission (OR 1.11; 95% CI 1.05 -1.18) and an active malignancy (OR 1.75; 95% CI 1.04-2.96) were independently associated with written TLDs (multivariate analysis, Table 4).

**Table 4.** Multivariate Analysis for Factors Independently Associated with TLD During ICU Stay in Patients with and without a Malignancy.

Covariate	OR^a^	95% CI^b^	P-value^c^
Age	1.03	1.01 -1.04	0.001*
Active malignancy	1.75	1.04-2.96	0.04*
CR^d^>1yr	1.48	0.72-3.05	0.29
Active malignancy + CR^d^	1.40	0.41-4.77	0.59
SOFA^e^ at ICU admission	1.11	1.05 -1.18	0.001*

^a^ OR; Odds ratio

^b^ CI; confidence interval

^c^ P- value; probability value, a p-value of < 0.05 was considered statistically significant, marked by an Asterisk *

^d^ CR; Complete remission

^e^ SOFA score; Sequential Organ Failure Assessment score (SOFA score)

### Secondary outcomes

Of the variables analyzed in the univariate binary logistic regression analysis of the study population, age (OR 1.05; 95% CI 1.01 -1.09), SOFA score at admission (OR 1.13; 95% CI 1.03 -1.25) and sepsis (OR 2.57, 95% CI 1.17-5.64) were associated with a written TLD during ICU stay (Table 5). After adjustment for the confounders, only age (OR 1.06; 95% CI 1.01 -1.11) remained associated with written TLDs (Table 6).

**Table 5.** Univariate Analysis: Factors Associated With TLD During ICU Stay in the Study Population.

Variable	Patient cases	Treatment limitation	OR^a^	95% CI^b^	P-value^c^
Age	–	–	1.05	1.01 -1.09	0.02*
Gender (male)	84 (67.2%)	24 (28.5%)	0.86	0.38 -1.94	0.72
Marital status-married (ref)	87 (69.6%)	25 (28.2%)	–	–	–
Without partner	30 (24%)	10 (33.3%)	1.24	0.51-3.02	0.64
Other	7 (5.6%)	2 (28.6%)	0.99	0.18-5.45	0.99
BMI^d^	–	–	1.00	0.92 -1.08	0.93
Comorbidity (CCI^e^)	–	–	0.98	0.80 -1.19	0.82
ECOG 0 (ref)	29 (23.2%)	7 (24.1%)	–	–	–
ECOG 1	35 (28%)	8 (22.9%)	0.93	0.29-2.97	0.90
ECOG 2	23 (18.4%)	10 (43.5%)	2.42	0.74-7.9	0.14
ECOG 3	25 (20%)	9 (36%)	1.77	0.54-5.75	0.34
ECOG 4	6 (4.8%)	2 (33.3%)	1.57	0.24-10.49	0.64
Solid (ref)	101 (80.8%)	29 (28.7%)	–	–	–
Hematological	21 (16.8%)	7 (33.3%)	1.24	0.46-3.39	0.67
Metastatic disease	33 (26.4%)	8 (24.2%)	0.91	0.34-2.49	0.86
Admission reason-medical (ref)	92 (73.6%)	27 (29.3%)	–	–	–
Elective surgery	10 (8%)	1 (10%)	0.27	0.03-2.22	0.22
Emergency surgery	20 (16%)	7 (35%)	1.30	0.47-3.60	0.62
Readmission	32 (25.6%)	9 (28.1%)	0.91	0.37-2.21	0.83
SOFA at ICU admission	–	–	1.13	1.03 -1.25	0.01*
Sepsis	54 (43.2%)	22 (40.7%)	2.57	1.17-5.64	0.02*

^a^ OR; Odds ratio

^b^ CI; confidence interval

^c^ P- value; probability value, a p-value of < 0.05 was considered statistically significant, marked by an Asterisk *

^d^ BMI; Body Mass Index

^e^ CCI; Carlson Comorbidity Index

^f^ ECOG; The Eastern Cooperative Oncology Group (ECOG) Performance Status.

^g^ SOFA; Sequential Organ Failure Assessment score (SOFA score)

**Table 6.** Multivariate Analysis: Factors Independently Associated with TLD During ICU Stay in the Study Population.

Covariate	OR^a^	95% CI^b^	P-value^c^
Age	1.06	1.01 -1.11	0.03*
Hematological malignancy	1.25	0.31-5.05	0.75
Admission reason-medical (ref)			
Elective surgery	0.61	0.06-6.37	0.68
Emergency surgery	1.78	0.46-6.88	0.40
SOFA at ICU admission^e^	1.14	0.98 -1.32	0.09
Sepsis	1.55	0.53-4.50	0.43
CCI	0.84	0.65 -1.09	0.20
ECOG 1	1.19	0.26-5.41	0.83
ECOG 2	4.19	0.89-19.75	0.07
ECOG 3	1.75	0.41-7.51	0.45
ECOG 4	1.48	0.17-12.92	0.72

^a^ OR; Odds ratio

^b^ CI; confidence interval

^c^ P- value; probability value, a p-value of < 0.05 was considered statistically significant, marked by an Asterisk *

^d^ ICU; Intensive Care Unit

^e^ SOFA; Sequential Organ Failure Assessment score (SOFA score)

Appendices B and C show that 30 patients (24%) of the study population were admitted to the ICU with a written TLD, and that having an active malignancy is associated independently with a written TLD before ICU admission (OR 3.60; 95% CI 2.17-5.97).

## Discussion

Treatment limitation decisions on the ICU can be challenging, especially in patients with a malignancy. Up-to-date literature regarding TLDs, and factors associated with TLDs, in critically ill patients with a malignancy admitted unplanned to the ICU is scarce. The aim of this study was to compare the incidence of written TLDs between patients with an active malignancy, patients with CR and patients without a malignancy during ICU stay. This study shows that having an active malignancy is independently associated with a higher risk of a written TLD during ICU stay, while patients with CR were not at risk for a higher incidence of TLDs.

The outcomes of patients with a malignancy after invasive life-saving treatments such as CPR and mechanical ventilation are potentially poor.^8,9,32,33^ The benefit of these treatments should be weighed against the possible burden of prolonged hospitalization and invasive treatment. It may be argued that our clinicians made TLDs in order to minimize inappropriate care in case of deterioration, especially when families indicate that quality of life was important for the patient.

On the other hand, some literature suggested that health care providers could display implicit “cognitive biases” toward patients,^34,35^ leading to a biased evaluation of patients based on certain characteristics. Biases toward patients with a malignancy among health care providers have been reported in literature.^36^ It could be argued that these biases might influence the perception of inappropriate care, leading to unintended discrimination of clinicians toward patients with a malignancy and subsequently more written TLDs in these patients. Literature suggests the existence of self-fulfilling prophecy (SFP) in medical decision making, especially in TLD and end-of-life decisions.^37^ Although this phenomenon is hypothetical and cannot be proven with objective studies regarding critically ill patients with a malignancy, from a psychological point of view, all clinicians should be aware of SFP. To avoid this pitfall, we organize a multidisciplinary meeting in our hospital once per day, including ICU physicians, physicians of the referring specialism, ICU nurses and if necessary, a clinical ethicist. Decisions concerning treatment limitations are made in this multidisciplinary meeting, and if possible, based on evidence in literature in our hospital.

Remarkably, the percentage of written TLD was almost a threefold higher in our study population than in the population of a recent study of a specialized Portuguese Cancer institute.^38^ In our study, the percentage of TLDs in patients without a malignancy was also higher than the percentage of written TLDs in ICU patients of large previous studies.^39,40^ Besides of differences in case-mix, it is well known that TLDs vary across countries, regions, hospitals, ICUs, and even among physicians.^38^ Religious beliefs, cultural backgrounds and the ethical climate of the ICU team can all influence such decisions.^1^ Therefore, the possibility exists that TLDs were actually made more often by our clinicians in similar patient’s circumstances. However, the association between having a malignancy and TLDs was found in previous studies as well.^41,42^

Our secondary objective was to identify factors independently associated with written TLDs in patients with a malignancy. Surprisingly, we found that only age was independently associated with a TLD, while comorbidity, gender and the ECOG performance status and severity of illness (SOFA score) were not associated. The influence of age on treatment-limitations decisions is consistent with other literature^41^ while an association with comorbidity, performance status, gender and severity of illness was found in other literature.^41,42^ The exact reasons for this difference remain unclear. However, similar to TLDs, ICU admission decisions vary across countries and regions, with religious beliefs and cultural backgrounds influencing ICU admissions. A difference in ICU admission considerations and subsequently a difference in case-mix may explain this difference with other studies.

In our study population, 75% did not have a treatment limitation before ICU admission, while the national guideline regarding critically ill patients with a malignancy states that the decision whether treatment limitations are appropriate should be made prior to an ICU admission. The absence of treatment limitations is acceptable in case of a good prognosis. Failure to discuss a treatment limitation due to difficulty with prognostication or due to inadequate or poor communication is reprehensible. This might lead to an inappropriate ICU admission and subsequently to a higher incidence of TLDs in patients with a malignancy during ICU admission. Early and proper education about impact and outcome is important to improve prognostication and communication of clinicians.^43–46^

All the TLD in our study population were made by the medical team in a multidisciplinary meeting, which is comparable to other literature.^47^ In Europe, TLDs are often made by the medical team. North American clinicians commonly apply standards or formal procedures and TLDs are more often made with family involvement due to the insurance system and the increase in litigation.^47^ However, caution regarding family involvement is recommended due to the following reasons: first, the knowledge of CPR in the general population is poor, mostly due to television medical dramas with poor representation of CPR and its outcome;^48^ second, family is often not able to adequately predict patient’s wishes^49^; last, a TLD made by family could cause a significant burden on the family and may result in anxiety or depression.^50^ Therefore a more paternalistic approach could be justified if the relevant factors for adequate decision making can be identified.

### Limitations and Strengths

First, the most important limitation of this study is the heterogeneous study population of this study, with different types of malignancies, differences in extensiveness of the malignancy and differences in cancer treatment before ICU admission. However, in our univariate analysis, no evidence of difference in TLD incidence was found between patients with a solid tumor and patients with a hematological malignancy and between non-metastatic disease and metastatic disease. Moreover, unintended discrimination by clinicians will be associated with the word cancer in general, not with a specific type of malignancy.

Second, physicians made a well-considered decision whether to admit the patient to the ICU, this could have influenced the incidence of TLDs before and during ICU.

Third, data were collected from a single institution, which can restrict generalizability.

Last, this was a retrospective study, and all the limitations of a retrospective review could be inherent in our study.

## Conclusions

This study shows that the presence of an underlying malignancy is independently associated with written TLDs during ICU stay. Patients with CR are not at risk of more written TLDs. TLDs are mostly made by the medical team. This is justifiable when made in order to minimize inappropriate care in case of deterioration. However, all clinicians should be aware of unintended discrimination toward patients with a malignancy. Therefore, TLDs should be made in a multidisciplinary meeting.

## Appendix

**Appendix A.** Numbers and Percentage Malignancy Types.

	Total study population *(n = 125)*
Type Solid malignancy	
Bladder carcinoma	4 (3.2%)
Cholangiocarcinoma	10 (8%)
Colorectal carcinoma	17 (13.6%)
Gastric carcinoma	3 (2.4%)
Hepatocellular carcinoma	3 (2.4%)
Larynx carcinoma	1 (0.8%)
Lung carcinoma	12 (9.6%)
Malignancy of central nerve system	4 (3.2%)
Melanoma	3 (2.4%)
Mesothelioma	3 (2.4%)
Neuroendocrine tumor	4 (3.2%)
Esophagus carcinoma	14 (11.2%)
Other	20 (16%)
Prostate carcinoma	5 (4%)
Sarcoma	1 (0.8%)
Type hematological malignancy	
Acute lymphoid leukemia	2 (1.6%)
Acute myeloid leukemia	6 (4.8%)
Non-Hodgkin Lymphoma	3 (2.4%)
Other	11 (8.8%)

The different types of the malignancies, note: some patients have more than 1 malignancy

## Appendix

**Appendix B.** Univariate Analysis for Factors Associated With TLD Before ICU Admission in Patients With and Without a Malignancy.

Variable	Patient cases	Treatment limitation	OR^a^	95% CI^b^	P-value^c^
No malignancy (ref)	825 (78.9%)	55 (6.7%)	–	–	–
Malignancy (active or CR^d^)	221 (21.1%)	49 (22.1%)	3.99	2.62-6.06	<0.001*
Active malignancy	125 (12.0%)	30 (24.0%)	4.42	2.70-7.24	<0.001*
CR^d^ < 1 yr	18 (1.7%)	1 (5.6%)	0.82	0.11-6.30	0.85
CR^d^>1yr	58 (5.5%)	11 (19%)	3.28	1.61-6.67	0.001*
No malignancy (ref)	825 (78.9%)	55 (6.7%)	–	–	–
Solid malignancy	172 (16.4%)	39 (22.7%)	4.11	2.62-6.44	<0.001*
Hematological malignancy	37 (3.5%)	7 (18.9%)	3.27	1.37-7.78	0.007*
Age	–	–	1.04	1.03 -1.06	<0.001*
Gender	661 (63.2%)	61 (9.2%)	0.81	0.54 -1.22	0.31

^a^ OR; Odds ratio

^b^ CI; confidence interval

^c^ P- value; probability value, a p-value of < 0.05 was considered statistically significant, marked by an Asterisk *

^d^ CR; Complete remission

## Appendix

**Appendix C.** Multivariate Analysis for Factors Independently Associated With TLD During ICU Stay in Patients With and Without a Malignancy.

Covariate	OR^a^	95% CI^b^	P-value^c^
Age	1.03	1.02 -1.05	<0.001*
Active malignancy	3.60	2.17-5.97	<0.001*

^a^ OR; Odds ratio

^b^ CI; confidence interval

^c^ P- value; probability value, a p-value of < 0.05 was considered statistically significant, marked by an Asterisk *

## Appendix

**Appendix D.** Characteristics and Outcome of the Study Population Without Treatment Limitations vs Those With Treatment Limitations.

	No TLD (n = 88)	TLD (n = 37)	p value^b^
Age^c^	65 [56-72]	68 [61-77]	0.02*
Male	60 (68.2%)	24 (64.9%)	0.72
BMI in kg/m^2 d, e^	24.0 {21.7-27.2}	23.8 {22.2-26.7}	0.93
Marital status			
Married	62 (70.5%)	25 (67.6%)	0.75
Without partner	20 (22.7%)	10 (27.0%)	0.61
CCI^c, f^	4 [3-6]	4 [2-6]	0.77
SOFA at ICU admission^c, g^	6 [4-9]	8 [6-11]	0.01*
ICU^h^ survival	69 (78.4%)	17 (45.9%)	<0.001*
Hospital survival	60 (68.2%)	11 (29.7%)	<0.001*
30 days survival	59 (67.8%)	9 (24.3%)	<0.001*
Length of stay^c, i^	2 [1-9]	7 [3-17]	0.003*
Withdrawal/withholding ICU^h^ treatment	19 (21.6%)	22 (59.5%)	<0.001*

^a^ DNR; Do-not-resuscitate

^b^ P- value; probability value, a p-value of < 0.05 was considered statistically significant, marked by an Asterisk *

^c^ Data are displayed as median with 25th and 75th percentile

^d^ BMI; Body Mass Index

^e^ A logarithmic transformation was performed, original data was not normally distributed

^f^ CCI; Carlson Comorbidity Index

^g^ SOFA; Sequential Organ Failure Assessment score (SOFA score)

^h^ ICU; Intensive Care Unit

^i^ In days
